# Handbook of Pain Surgery

**DOI:** 10.1055/s-0038-1669392

**Published:** 2018-08-13

**Authors:** J. Bahm

**Affiliations:** 1 Euregio Reconstructive Microsurgery Unit, Franziskus Hospital, Aachen, Germany

**Handbook of Pain Surgery by Kim J. Burchiel [Thieme 2018]**

Pain might be a terrible experience for the patient and still remains difficult to be treated. Pain surgery thus is of great interest for all therapists encountering pain patients.

The present handbook gives a clear overview of common techniques and procedures with a clearly structured text and a lot of figures.

The first chapter on approach to the patient with chronic pain sets the frame: take the time to listen to the patient, his story, and pain characteristics. How demanding this may be!

In section II, several authors develop the stimulation techniques addressing the peripheral nerves or spinal cord and intrathecal drug administration before focusing on the surgical options in trigeminal neuralgia.

The last section is on destructive procedures addressing the myelon to interfere with pain-transmitting pathways.

This concise handbook is a must for everyone who deals with patients experiencing severe, chronic, especially neuropathic pain, either general practitioners, anesthetists, neurologists or neurosurgeons, and upper limb surgeons. Even if we are not performing these procedures ourselves, we need to be aware of *all* existing techniques and evaluated procedures, to bring these into an inclusive discussion with the patient seeking our advice.

This handbook gives a clear and well-documented overview and thus allows a better, more complete, more elaborated multidisciplinary approach to chronic pain, which does not respond sufficiently to analgesic regimens.

Of course we should always bear in mind that the chronic pain needs to be addressed not only by medication and surgery, but the whole suffering individual should be considered including help for his/her daily living, psychology, emotional coping with pain, and still mandatory life project.

